# Insights into the role of N6-methyladenosine (m6A) in plant-virus interactions

**DOI:** 10.1128/jvi.01598-24

**Published:** 2024-11-21

**Authors:** Nicola Secco, Arsheed H. Sheikh, Heribert Hirt

**Affiliations:** 1 Biological and Environmental Science and Engineering Division (BESE), King Abdullah University of Science and Technology (KAUST) 127355 , Thuwal, Saudi Arabia; 2 Plant-Microbe Interactions, Department of Biology, Utrecht University 98808 , Utrecht, the Netherlands; Indiana University Bloomington, Bloomington, Indiana, USA

**Keywords:** RNA modification, m6A, plant virus

## Abstract

N6-methyladenosine (m6A) is a common and dynamic epitranscriptomic modification in eukaryotic RNAs, affecting stability, splicing, translation, and degradation. Recent technological advancements have revealed the complex nature of m6A modifications, highlighting their importance in plant and animal species. The m6A modification is a reversible process, with “writers” depositing methylation, “erasers” demethylating it, and “reader” proteins recognizing m6A and executing various biological functions. Studying the relationship between m6A methylation and viral infection is crucial. Animal viruses, including retroviruses, RNA viruses, and DNA viruses, often employ the host’s m6A machinery to replicate or avoid immune responses. In plant viruses, host methyltransferases or demethylases can stabilize or degrade viral RNA, depending on the virus-host interaction. Additionally, viral infections can modify the host’s m6A machinery, impacting the viral life cycle. This review examines the role of m6A modifications in plant viral pathogenesis, focussing on RNA viruses infecting crops like alfalfa, turnip, wheat, rice, and potato. Understanding the role of m6A in virus-host interactions can aid in studying plant viral disease development and discovering novel antiviral targets for crop protection. In this review, we summarize current information on m6A in RNA biology, focussing on its function in viral infections and plant-virus interactions.

## INTRODUCTION

RNA methylation is a common chemical modification that regulates multiple aspects of RNA metabolism (1), including stability (2), splicing (3), translation (4), and degradation (5). The enzymes involved in this specific pathway are classified into three groups: writers, readers, and erasers.

### Writers

m6A is the most common and dynamic RNA modification where a methyl group is deposited on the N6 position of an adenosine base by a methyltransferase enzyme known as writer. Several adenosine methyl transferases have been identified in mammals and other organisms, each with different substrate preferences and different structures. 18S rRNA and 28S rRNA are prevalently methylated by METTL5 and ZCCHC4 (6, 7). The *METTL5* gene was identified by screening for proteins harboring adenine methylase domains. Although being an adenosine methyl transferase, METTL5 structure differs from the other known METTL enzymes. The heterodimer of METTL5/TRM112 is recruited during ribosome biogenesis and targets 18S rRNA. ZCCHC4 is a zinc finger protein that catalyzes 28S rRNA m6A methylation (6). ZCCHC4 is catalytically active *in vitro* and in *in vivo* without any other cofactor, and ZCCHC4 knockout completely abolishes 28S adenosine methylation at A4220 of 28S rRNA (6).

m6Am is incorporated on 2′-O-methyl adenosine (Am) on the 5’ end of mRNAs. This methylation is catalyzed by the PCIF1 (CAPAM) methyltransferase (8), through recognition of 7-methylguanosine (m7G/cap), and Am also needs to be present on the transcript for the m6A methylation to occur. m6Am is catalyzed co-transcriptionally, and although it does not affect transcript stability, it enhances transcript translation (8).

The most prominent adenosine methyltransferase in mRNA and lncRNA is a multienzymatic complex with a mass of 1 MDa in metazoan organisms. The complex catalytic core is composed of the methyltransferase METTL3 and its cofactor METTL14 (9). Although the latter does not exhibit detectable methyltransferase activity, mutations in *mettl14* lead to embryonic lethality, similar to *mettl3* (10, 11). In addition, post-transcrptional gene silencing (PTGS) of *METTL14* leads to a notable reduction in m6A, although METTL3 alone is capable of catalyzing the reaction *in vitro* (11). The catalytic activity of METTL3 resides in the amino acids D337, D395, N539, and E532, which are conserved in both *METTL3* and its orthologs, but not in METTL14 (12). In plants, the orthologs of *METTL14* and *METTL3* are *MTB* and *MTA*, respectively (13, 14).

MTA shares similar traits with its metazoan equivalent and operates as an active methyltransferase enzyme (13). Although being able to form homodimers in *Arabidopsis*, it is not clear whether the homodimer forms an active methyltransferase enzyme (14). MTB, despite not having methyltransferase activity in *Arabidopsis*, influences MTA localization and catalytic activity (14, 15). Nevertheless, MTB may exhibit catalytic activity in other plant species (16).

WILMS' TUMOR 1-ASSOCIATED PROTEIN (WTAP) has a role in increasing the stability of the METTL3 and METTL14 heterodimers, as well as aiding in the positioning of the resultant complex near nuclear speckles (17). The plant ortholog of WTAP has been identified as *FKBP12 Interacting Protein 37* (*FIP37*) (14, 15).

VIRMA, a homolog of the sex-determining Drosophila *VIRILIZER* (18, 19), is another protein in the complex. VIRMA is the biggest protein in the methyltransferase complex and recruits the METTL3/METTL14/WTAP catalytic core (20). Although its precise role remains elusive, it is involved in several human diseases, such as tumorigenesis and heart diseases (21). *VIRMA*’s ortholog in *Arabidopsis* is *VIR1*, which functions primarily as a scaffolding protein (14, 15, 17, 22).

**Fig 1:**
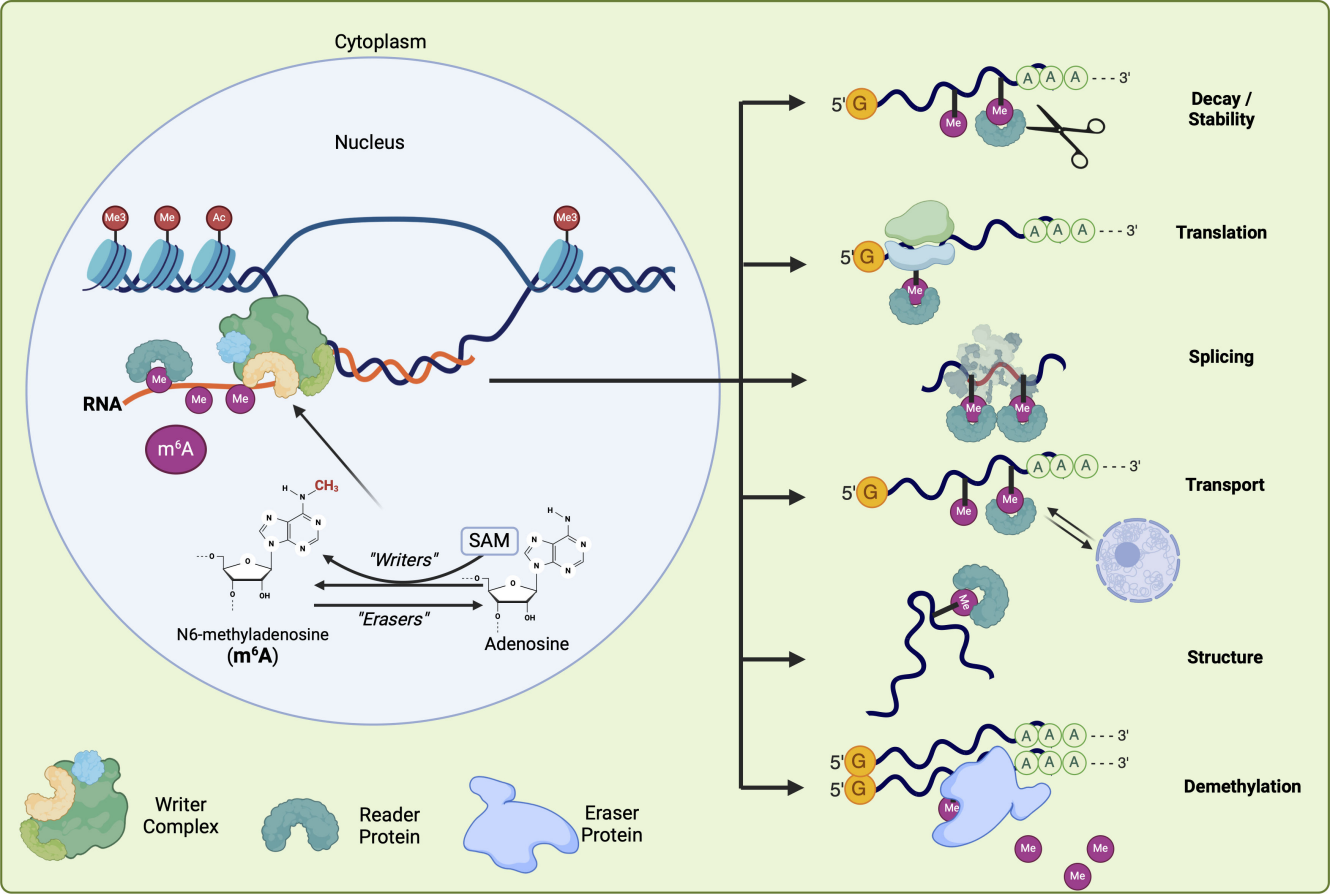
Schematic representation of the methylation process: most eukaryotic mRNAs and lcRNAs are methylated in the nucleus, costranscriptionally. Although some reader proteins are nuclear localized and can exercise their activity directly in the nucleus, most reader proteins are cytoplasmic and need the m6A labeled RNA to exit the nucleus. Eraser proteins are found in both the nucleus and cytoplasm, and they are shown only in the latter since most of the erasers described in this work are cytoplasmic.

HAKAI, an E3-ubiquitin ligase, is present in both plants and animals. The specific role of *HAKAI* in the methyltransferase complex is still unclear. *hakai* mutants display a decrease in m6A levels in *Arabidopsis* and metazoans, suggesting its probable role in m6A deposition (Fig. 1) (14, 15, 23, 24).

### Readers

m6A stabilizes single-stranded configurations by impairing RNA A helix formation due to stereochemical hindrance (25, 26). However, the main downstream effects are caused by a group of mediators, known as readers, that have the ability to recognize and bind to the m6A modification in transcripts. Reader proteins contain the YT-521 homology (YTH) domain, which is crucial for recognizing and binding to the m6A modification (27). This domain is characterized by two tryptophan and a leucine residue, which form a hydrophobic pocket that stabilizes the interaction with m6A. Additionally, a complex of two asparagine and a serine residue facilitates the formation of hydrogen bonds with the adenosine base, enhancing the specificity of this recognition (25). The YTH has a 20–50 times higher affinity for m6A compared with adenosine but has a very poor affinity for RNA molecules (25). Although the other domains of the YTH proteins vary among different species, this domain is preserved in both metazoan and plant species (27). In *Homo sapiens*, the YTHDC1/2 proteins are found in the nucleus, whereas YTHDF1-3 are located in the cytoplasm (28). Based on the presence of YTH domain, 13 genes in *Arabidopsis* were identified and designated as *EVOLUTIONARY CONSERVED C-TERMINAL REGION* (*ECT*s) (29). Most of the ECT1-11 proteins are homologous to human YTHDFs and are probably located in the cytoplasm. In contrast, ECT12/13 exhibit a strong similarity with YTHDCs, indicating that they are likely located in the nucleus (29). Another reader protein showing strong homology to YTHDCs is CLEAVAGE and POLYADENYLATION SPECIFICITY FACTOR 30 (CPSF30) (30). CPSF30 regulates several processes such as programmed cell death, immunity, and signaling in *Arabidopsis* (30–33). ECT2 is one of the most studied members of this group. *Arabidopsis ect2* mutants have phenotypic traits that resemble those of other mutants in the writer complex, showing an increased level of trichome branching (34). The majority of m6A binding sites of ECT2 are located near the 3’ untranslated region (UTR) of mRNAs and have a role in regulating transcript stability. The results indicate that ECT2 acts as an RNA stabilizer, in contrast to YTHDF2, which has been previously shown to facilitate target degradation (29). ECT2 is shown to bind the URU[m6A]Y motif as identified by formaldehyde iCLIP (FA-CLIP) sequencing (29, 34, 35). The cascade of events that follows m6A recognition by reader proteins is summarized in Fig. 1.

### Erasers

In metazoans, m6A demethylation is performed by two proteins: fat mass and obesity-associated (FTO) and ALKBH5 (36). ALKHB family homologs were initially identified as primary erasers because they possess a nonheme Fe(II) *α*-ketoglutarate-dependent dioxygenase domain (36). In plants, 13 members of the *ALKHB* family have been identified, including demethylases ALKHB9B and ALKHB10B have been verified *in vivo*, whereas only the latter has been shown to have demethylase activity *in vitro* (37, 38). Erasing m6A can lead to changes in RNA maturation, stability, transportation, translation, secondary structures, and modification of lncRNA activity (Fig. 1) (39–45).

### m6A and viral infections

Viruses and virions replicate using the host’s metabolic machinery, which often leads to alterations in cell metabolism and cell death. Metazoans can be infected by double-stranded (ds) or single-stranded (ss) DNA or RNA viruses. Positive strand (+) and negative strand (−) ssRNA viruses and retroviruses are known to be regulated via m6A. In HIV, several m6A peaks have been identified in the viral gRNA. Those that have been confirmed to interact with YTHDFs proteins are primarily located toward the end of the viral transcript, spanning from the env region to the 3’UTR of the viral gRNA. The interaction between YTHDFs and m6A-modified viral RNA affects transcript stability, promoting efficient viral replication (46–49). Similarly, m6A peaks were found in the -ssRNA influenza A virus (IAV), which can bind YTHDF proteins for efficient pathogenicity and replication rate (50). On the other hand, for +ssRNA viruses like hepatitis C virus (HCV) and zika virus (ZIKV), the methyltransferase complex inhibits these viruses (51, 52). In HCV, viral m6A peaks align with the ENV protein binding motif. Thus, YTHDFs readers compete with ENV for gRNA binding, leading to the degradation of viral transcripts due to reduced virus assembly (51). YTHDF2 has also been reported to destabilize ZIKV viral transcripts (52). Taken together, in a host-pathogen-specific relationship, methylation of viral genomes or transcripts can influence viral replication at multiple levels, operating as a defense mechanism or promoting viral proliferation. More importantly, despite the genome type and replication mechanism, transcripts of diverse viruses are significantly influenced by m6A in animals.

## m6A METHYLATION: A CRUCIAL REGULATOR OF PLANT-VIRUS INTERACTIONS AND VIRAL PATHOGENESIS

Recent studies in both model and crop plants have shed light on the critical role of the m6A machinery in regulating plant viral infections. Some of the important case studies are discussed in detail below.

### AMV exploits ALKBH9B to evade plant m6A-mediated defense mechanisms

**Fig 2:**
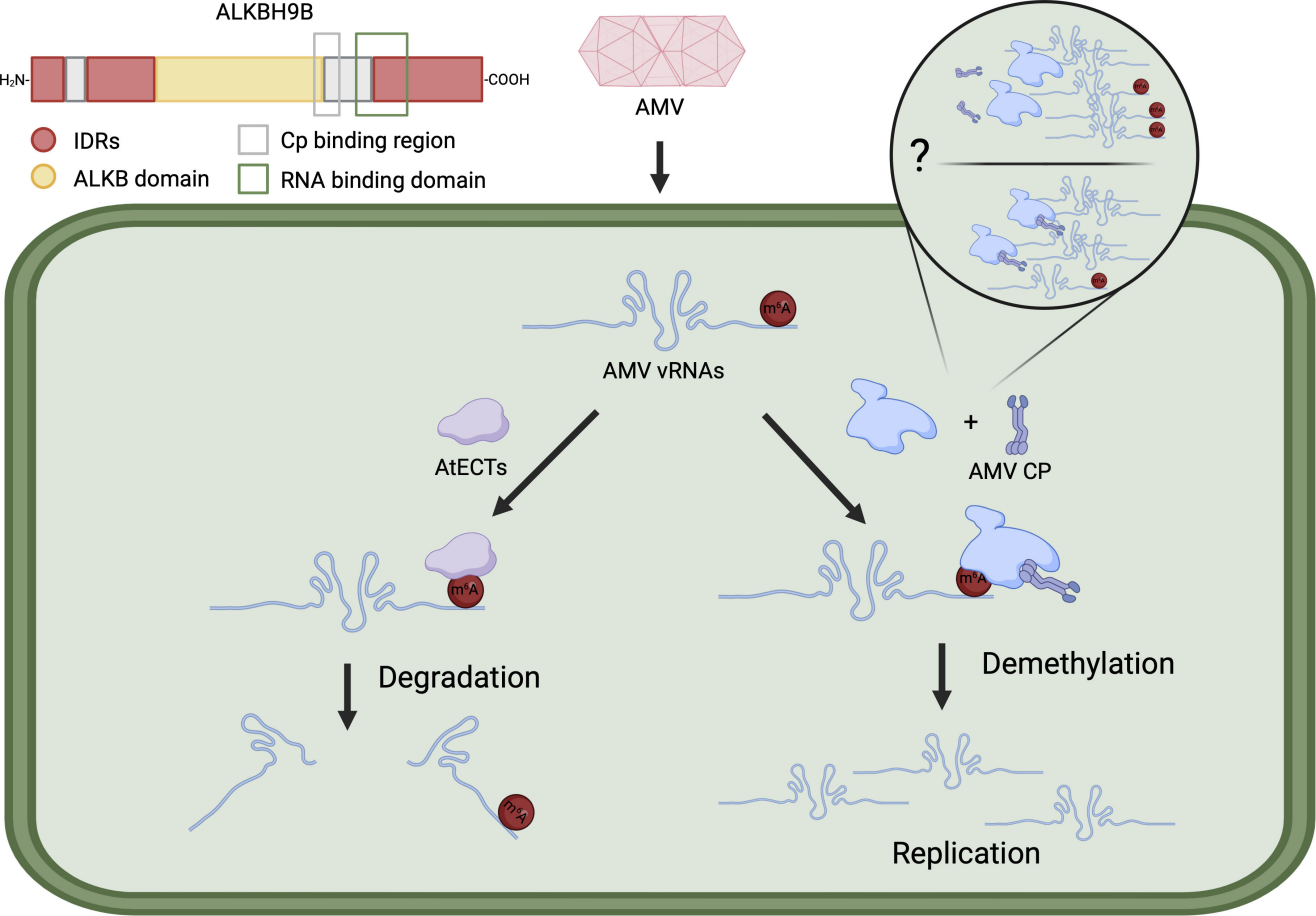
AMV vRNAs contain several m6A peaks. Host ECT proteins recognize and bind m6A marks on vRNAs, leading them to degradation. Conversely, the host eraser protein ALKBH9B interacts with the viral coat protein (CP) and demethylates vRNAs, thereby promoting viral replication. The actual impact of ALKBH9B and Cp interaction remains unclear.

The infection of alfalfa mosaic virus (AMV), a +ssRNA plant virus belonging to the *Bromoviridae* family is regulated by the m6A machinery (38). The plant demethylase ALKBH9B is present in processing bodies (p-bodies) and involved in RNA degradation through nonsense-mediated decay (NMD) and silencing of both endogenous mRNA and viral transcripts. ALKBH9B is entirely cytoplasmic, and its activity was verified *in vitro* (38). The *alkbh9b* mutants in *Arabidopsis* displayed a reduced buildup of AMV. Subsequent examinations revealed many methylation peaks in the viral genome (38). This was the first complete report of an m6A-dependent viral modification *in planta*, where m6A negatively impacted virus replication. In cucumber mosaic virus (CMV), another member of the *Bromoviridae* family, specific methylation peaks were also found. However, there were no noticeable differences in CMV methylation peaks found when comparing *alkbh9b* and wild-type *Arabidopsis* plants (38). The AMV viral titer was restored in *alkbh9b* mutants with additional mutations in *ECT2*, *ECT3*, and *ECT5*. The necessity for m6A-mediated defense may stem from the fact that AMV and related viruses exhibit lower susceptibility to siRNA-mediated defense compared with other +ssRNA viruses. ECTs were demonstrated to play a pivotal role in regulating the decay of AMV viral RNA (vRNA), whereas mutations in methyltransferase components like VIR1 had negligible impact. MTA, MTB, FIP37, and HAKAI protein levels are directly related to *VIR1* expression (15). This suggests that there may be a methyltransferase whose activity is independent of *VIR1* expression or that the recognition by ECTs plays a more significant role in influencing viral metabolism than the actual levels of methylation. Unlike animal YTHDF2, ECT2/3/5 enhances the stability of target transcripts but has the opposite function in AMV vRNA, represented in Fig. 2 (53, 54). AtALKBH9B maintains its RNA-binding ability specifically within the amino acid range of 427 to 467, which are expected to form intrinsically disordered regions (IDRs). The ALKB-*like* domain is situated between the N-terminus IDR and the C-terminus IDR. Deletions in residues before or after the ALKB-*like* domain lead to the absence of P-body localization. Moreover, the amino acids at the C-terminus of the ALKB-*like* domain are crucial for interacting with AMV-CP, likely reflecting a viral adaptation to recruit ALKBH9B to its viral RNA (55, 56). AMV exhibits a higher tendency to interact with ALKBH9B due to its high quantity and cytoplasmic localization. Interestingly, *alkbh9b* plants exhibit hindered viral cell-to-cell mobility, particularly impacting movement in plant roots where AMV cannot colonize the phloem due to its reduced mobility, likely caused by the high m6A abundance in the viral RNA, leading to exclusion by plasmodesmata monitoring mechanisms. This study consolidates *ALKBH9B*’s significant role in plant-virus interactions (53, 54).

### Horizontal gene transfer and functional variability of viral ALKB-like domains in plant viruses

Some viruses encode ALKB-*like* domains (vALKB) in their genomes, including several members of the *Flexiviridae* family. Interestingly, ALKB-*like* domains are not very conserved and show substantial variability between the members. Phylogenetic analysis showed that these sequences have been acquired by horizontal gene transfer by a *Flexiviridae* progenitor and then passed down to the family clades or even to separate viral families (57–59). *Flexiviridae* infect primarily perennial plants, and most of the family members that possess a functional ALKB-like domain preferably infect the phloem (57). Nonfunctional ALKB-*like* domains are found in viruses that infect seasonal plants, suggesting that these domains are no longer necessary for virus replication (57, 59, 60).

### Mechanisms of host m6A-mediated defense evasion by plant viruses

**Fig 3:**
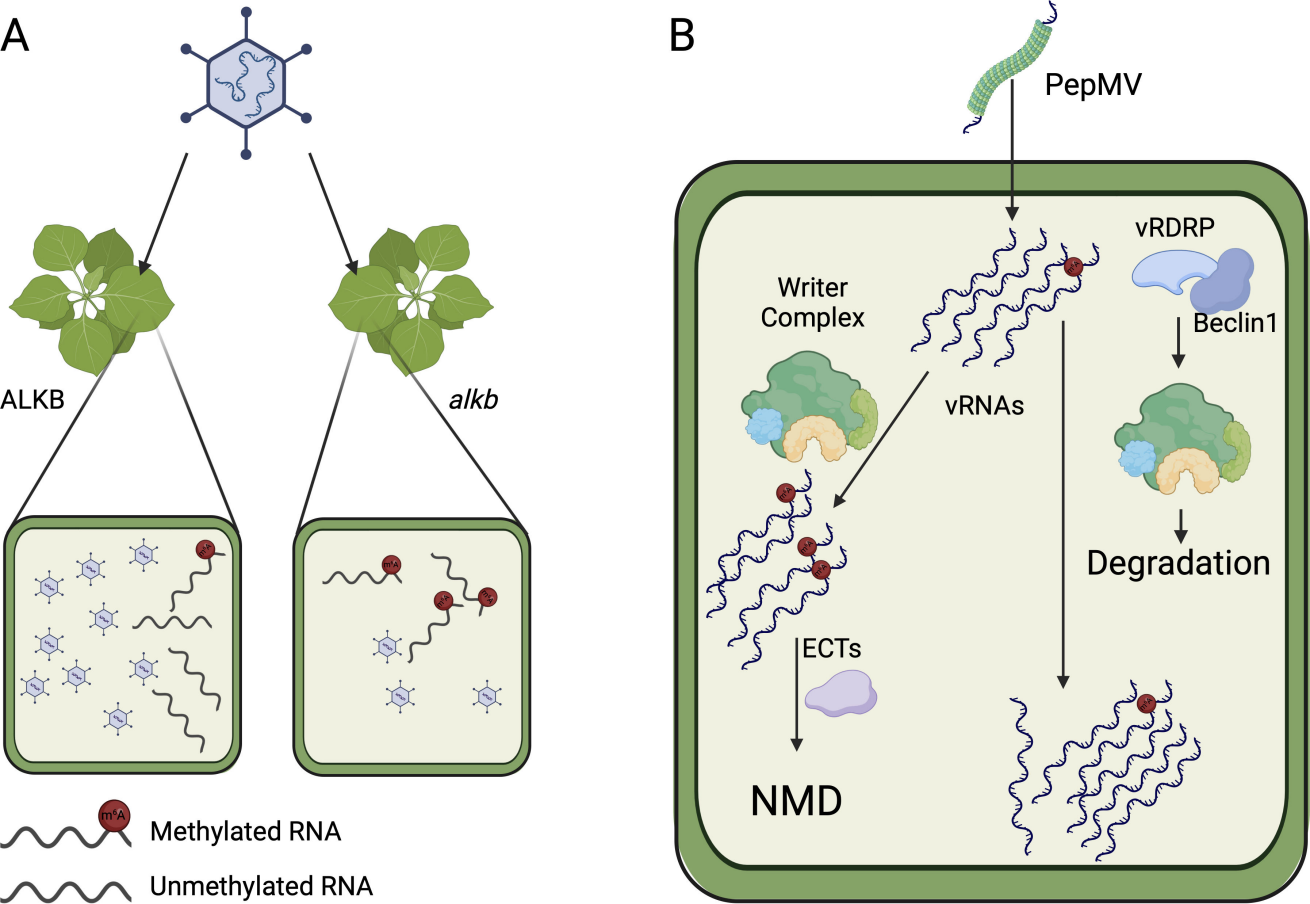
Mechanisms used by viruses to evade m6A-based defenses. (A) TMV, PPV, and PVY cause host m6A levels to drop significantly during the infection. Downregulation or mutations in the host ALKB enzymes cause a reduction in viral titer and impede viral replication and proliferation. (B) Schematic representation of PepMV strategy to evade plant m6A modifications by directing HAKAI toward autophagic degradation.

Despite the absence of ALKB-*like* domains, several viruses belonging to different families are capable of evading the host m6A defense by either promoting their transcript demethylation or by impairing the host methyltransferase ability: potato virus y (PVY) and plum pox virus (PPV) decrease the m6A titer in infected hosts. PVY and PPV have methylation peaks near their 3’ untranslated regions (3’UTRs). Suppressing *NtALKBH9B* homologs significantly decreased the viral titer in PPV-infected samples, with similar outcomes for PVY (59). Using ultra-high-performance liquid chromatography coupled with high-resolution mass spectrometry (UHPLC-HR-MS/MS), it was demonstrated that tobacco mosaic virus (TMV) decreases the total m6A levels in *N. tabacum*, accompanied by elevated *NtALKBH5* expression, directly facilitating TMV infection (61). This finding corroborates previous studies showing that +ssRNA viruses suppress host methyltransferase activity to promote replication and the lytic cycle as depicted in Fig. 3 (61).

Pepino mosaic virus (PepMV), an *Alphaflexiviridae* family member, poses a significant threat to solanaceous plants. Overexpression of *NbMTA* and *NbHAKAI* led to a substantial decrease in PepMV viral titer and increased methylation in native transcripts while silencing of *NbECT2A*, *NbECT2B*, and *NbECT2C* enhanced the PepMV-induced phenotype (62). NbECTs interact with proteins involved in NMD, targeting viral transcripts to processing bodies for breakdown (63). Interestingly, PepMV vRNA is guided toward NMD by the cellular m6A machinery, whereas PepMV RNA-dependent RNA Polymerase (RDRP) interacts with SlHAKAI, driving SlHAKAI toward autophagic degradation by connecting with SlBeclin1 in the cytoplasm as shown in Fig. 3. PepMV decreases the methylation of its vRNAs by interfering with the host-writer complex. Autophagy, typically linked to viral infections as a protective process, may have a direct link with methylation in viral defense (62).

### A single polymorphism separates viral resistance from viral susceptibility in wheat

**Fig 4:**
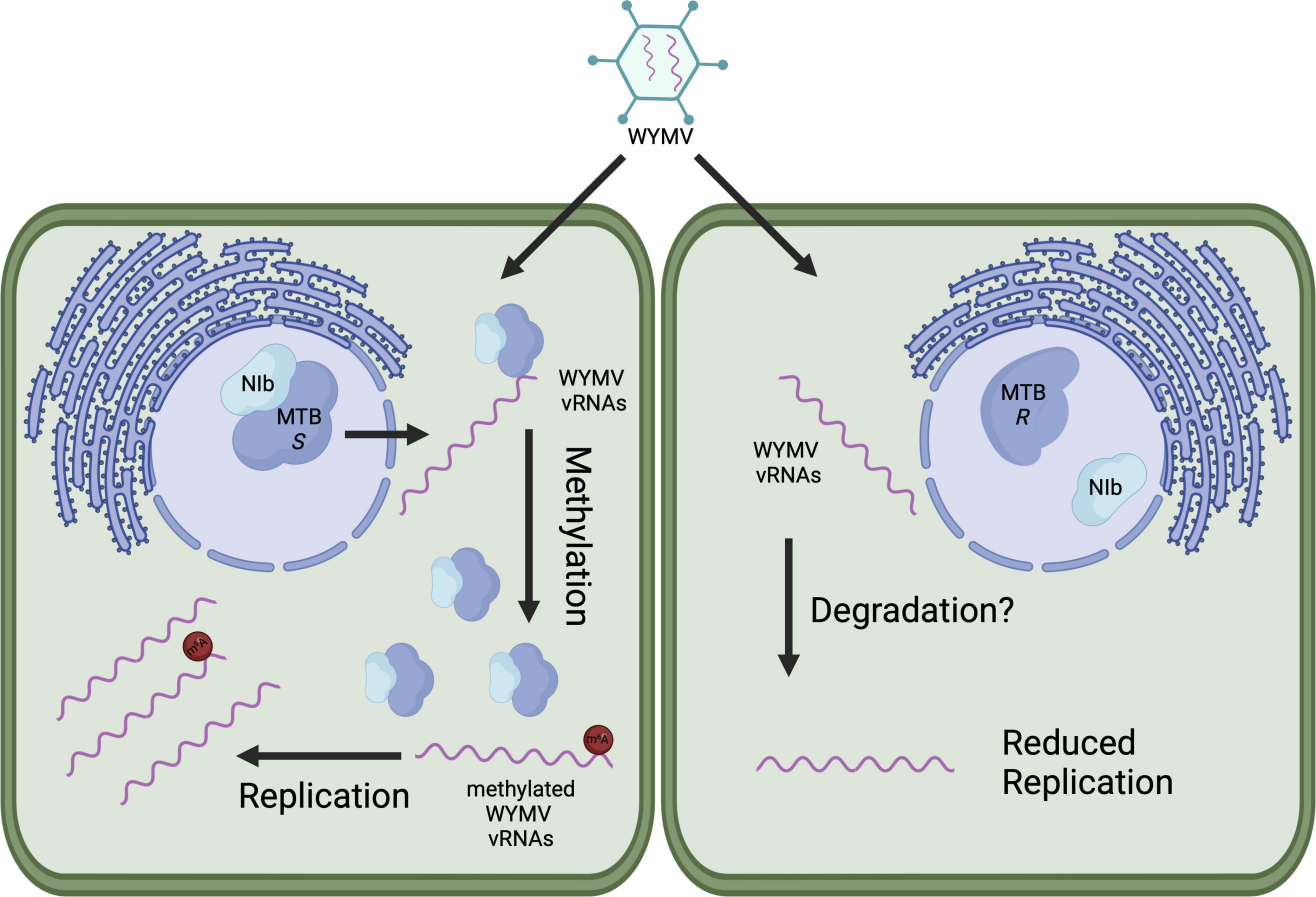
WYMV replication and virulence are positively influenced by host m6A methylation. In susceptible wheat varieties, the viral NIb protein binds to the host MTB protein, causing its relocalization to the cytoplasm, where it enhances vRNA methylation, promoting viral replication. In contrast, this interaction is disrupted in resistant wheat varieties resulting in significantly reduced vRNA methylation. This difference is attributed to a SNP at position 176, distinguishing sensitive ("*S*") from resistant ("*R*") wheat varieties.

A role of m6A was demonstrated in the immune response of two distinct wheat varieties, denoted as either wheat-resistant variety (WRV) or wheat-sensitive variety (WSV), to wheat yellow mosaic virus (WYMV) (64). Gene expression and m6A profiles were significantly different between these varieties when comparing infected and healthy plants. Of all virus-induced differentially regulated genes, almost half showed a negative correlation with m6A methylation levels, whereas the other half had a positive correlation (64). Interestingly, among these genes, two methyltransferase-related genes namely *TaFIP37* and *TaALKBH29B* showed a significant misregulation after WYMV infection. Similar outcomes were documented in rice plants, corroborating the regulatory function of m6A in the context of plant viral infections (64, 65). Wheat resilience to WYMV was further analyzed for the purpose of identifying the specific gene variants, which were referred to as sensitivity genes (S genes). Interestingly, component of wheat m6A writer complex, *MTB* was identified as one of the S genes. Overexpressing of the primary S gene *TaMTB* in wheat led to increased susceptibility to WYMV, whereas knocking down *TaMTB* expression enhanced the cultivars’ resilience to the virus. Although *E. coli*-purified TaMTB did not exhibit methyltransferase activity, plant-purified TaMTB was able to methylate RNA targets (16). However, a notable methylation peak was detected in WYMV RNA1, prompting an investigation of the mechanism by which this virus undergoes methylation, considering its replication takes place in the cytoplasm. The translocation of TaMTB to the cytoplasm was observed following the expression of NIb (WYMV RDRP). Specifically, TaMTB gets delocalized in NIb-P1-induced inclusion bodies, most likely to be incorporated in WYMV viral replicating centers. Of the 243 wheat genomes analyzed, only two TaMTB alleles were found which differed by a single nucleotide polymorphism (SNP) at position 176 (SNP176C, SNP176A) (16, 64). SNP176C exhibited greater affinity for NIb and had consequently higher methylation levels in WYMV RNA1 as summarized in Fig. 4. Remarkably, this polymorphism does not impact the productivity of wheat, making it an excellent candidate for selective breeding (16).

### Insect vector influence on viral m6A methylation and plant-host responses

The majority of plant viruses rely on insect vectors to infect new hosts. The Small Brown Planthopper (SBPH) serves as the vector for rice black-streaked dwarf virus (RBSDV), which has a double-stranded RNA genome. Using RNA interference against *LsMETTL3* and *LsMETTL14* to downregulate insect methyltransferases leads to an increase in the viral titer in SBPHs insects. RBSDV methylation levels in the insect vector were inversely correlated with its virulence, suggesting insect vectors may use methylation to regulate virus replication (66). Although viral methylation may occur in the insect vector before it infects the plant host, RBSDV and rice stripe virus (RSV) were able to dramatically disrupt the plant’s m6A spectrum after infection (65). Notably, although some of the m6A peaks were shared upon both virus infections, certain peaks were exclusively found in each virus. This indicates that the cell methylome exhibits distinct responses depending on the viral infection. Among the common methylation peaks, *OsAGO18* and *OsAGO2* were differentially methylated, and this impacted their expression levels, suggesting a direct connection between m6A and the RNA interference response (65).

### Virus infections can alter hormone signaling through m6A methylation

Hormone signaling plays a crucial role in plant communication. The primary hormones involved in rice plant growth and development include jasmonic acid (JA), salicylic acid (SA), abscisic acid (ABA), auxin, ethylene, brassinosteroids (BR), and cytokinins. Transcripts associated with these signaling pathways had m6A peaks, and their expression and methylation levels were notably modulated by viral infection (65). The GO term analysis of the rice transcriptome showed enrichment of signal transduction, hormone signaling, and sugar and amino acid metabolism. Of these, 21 transcripts exhibited methylation peaks following RBSDV and RSV infections. These modifications often occur in genes with low expression and influence alternative polyadenylation (APA) sites, leading to the downregulation of antiviral genes. By altering the plant’s polyadenylation landscape, viruses can manipulate host gene expression to suppress defenses. This balance between m6A modifications and APA allows viruses to adapt and thrive in the host, highlighting m6A’s crucial role in virus-host interactions (65).

### Role of nuclear m6A machinery in regulating cytosolic RNA viruses

Given that the majority of plant viruses are RNA-based, their metabolism is largely confined to the cytoplasm and organelle compartments. Several RNA viruses have been found to undergo direct methylation in their transcripts or interact with the methylome complex, often at multiple levels (38, 53, 54, 62, 63, 65). The majority of RNA-based viruses exhibit a distinct replication cycle that does not entail nuclear infiltration. Instead, their replication and assembly primarily occur within the Golgi, mitochondria, chloroplast, and endoplasmic reticulum (ER). The evidence of methylation in RNA viruses suggests a potential interaction between these viruses and the writer complex, which is predominantly thought to reside in the nucleus (67). The explanation for this observation was initially tackled by observing that a certain degree of METTL3 or MTA can be detected in the cytoplasm (11, 14, 15, 67). Also, viral replicating units may either sequester or undergo methylation prior to the enzyme’s arrival at its ultimate destination. Nevertheless, different studies have shown that modifications in the writer complex cannot only affect methylation efficacy but also induce alterations in the localization of its constituents (15, 68). In *Arabidopsis*, it has been observed that upon the implementation of miRNA-based PTGS on *MTA*, the MTB protein translocates from the nucleoplasm to the cytoplasm (15). This phenomenon has also been documented for all the other constituents of the *Arabidopsis* writer complex, with the exception of HAKAI (15). This may be the result of a virally evolved system designed to destabilize the m6A apparatus to promote viral replication or shield viral transcripts from host defenses.

Table 1 summarizes the reported m6A-virus interactions. Viruses are listed in the same order they are presented in the main text. The type of interaction is defined negative (−), positive (+), or unclear (*), depending on the effect that vRNA methylation or host-altered methylation has on the virus (when demethylation favors viral metabolism or the virus reduces host m6A levels it is considered negative).

**TABLE 1:** m6A impact on viruses^*a*^

Virus	m6A impact on virus	Family	Genome	Description
Alfalfa mosaic virus (AMV)	–	*Bromoviridae*	+ssRNA	AMV is negatively regulated by AtECTs and recruits AtALKBH9B through direct interaction with CP (38, 53–56)
Cucumber mosaic virus (CMV)	*	*Bromoviridae*	+ssRNA	CMV vRNAs are methylated in *Arabidopsis*. However, CMV replication is not affected by ALKBH9B (38)
Plum pox virus (PPV)	–	*Potyviridae*	+ssRNA	PPV decreases plant m6A levels and knockdown of NbALKBH9 caused a reduction in the viral titer (59)
Potato virus y (PVY)	–	*Potyviridae*	+ssRNA	PVY decreases plant m6A levels and knockdown of NbALKBH9 caused a reduction in the viral titer (59)
Tobacco mosaic virus (TMV)	–	*Virgaviridae*	+ssRNA	TMV infection increases *NbALKBH5* expression and reduces m6A level in *N. tabacum* (61)
Pepino mosaic virus (PepMV)	–	*Alphaflexiviridae*	+ssRNA	PepMV counters host m6A NMD antiviral defense by directing HAKAI toward autophagic degradation (62, 63)
Wheat yellow mosaic virus (WYMV)	+	*Potyviridae*	+ssRNA	WYMV recruits host MTB into the cytoplasm located VRCs through the interaction with NIb (16, 64)
Rice black-streaked dwarf virus (RBSDV)	–	*Sedoreoviridae*	dsRNA	RBSDV is methylated by the aphid vector and interferes with aphid m6A deposition and PTGS; *in planta* RBSDV alters m6A, resulting in hormonal imbalance and altered polyadenylation (65, 66)
Rice stripe virus (RSV)	*	*Phenuiviridae*	-ssRNA	RSV alters m6 A resulting in hormonal imbalance and altered polyadenylation (65, 66)

^*a*^ Table summarizing the reported m6A-virus interactions. Viruses are listed in the same order as presented in the main text. The type of interaction is defined as negative (−), positive (+), or unclear (*), depending on the effect that vRNA methylation or host-altered methylation has on the virus (when demethylation favors viral metabolism or the virus reduces host m6A levels. It is considered negative).

## CO-EVOLUTION OF m6A MACHINERY COMPONENTS IN VIRAL GENOMES

Viruses and their plant hosts are locked in a continuous evolutionary arms race, where each exerts intense selective pressure on the other. This dynamic interaction drives the host to develop new antiviral defenses while pushing viruses to evolve strategies to evade or suppress these defenses. The possibility of a co-evolutionary mechanism is reinforced by the presence of demethylase domains in certain viral proteins: conserved ALKBH-like domains are present in the viral proteins of the *Flexiviridae* and *Potyviridae* families, in some instances originating from distinct events (57, 59, 60, Table 2). These domains were incorporated into viral genomes relatively recently, coinciding with the increased use of pesticides and other chemicals in agriculture that induce DNA or RNA methylation. It is not clear if these motifs were introduced in the viral genome through anthropogenic activities or to counter m6A-mediated plant defense (57, 60). The timing of the appearance of these motifs and their preservation in several viral genomes likely reflects a common selective pressure on viruses that infect perennial plants with chronic infections (57, 59, 60). Multiplicity of active viral infections is also quite common in perennial plants, which likely explains the rapid and widespread diffusion of viral ALKB-like (vALKB) domains within viral genomes over a relatively short period. The fact that these motifs can be found selectively either in the virus replicase cistron (Flexivirus, Picornavirus, and Closterovirus), which suggests a co-transcriptional demethylation activity, or in the P1 protein (Potyviruses), which indicates a post-transcriptional effect, seems to be family-specific and might indicate two different ways to use the same tool (57–60). This suggests that the host methylome exerts a selection pressure on the evolution of these viruses. It remains unclear whether the positioning of the *ALKB-like* domain in the viral genome plays a role in demethylating viral RNAs or if it interferes with the host’s DNA and RNA methylation processes. vALKB domains have been shown to have a higher affinity for ssRNA or dsRNA, which diminishes the likelihood of these domains playing a role in interfering with genomic DNA methylation (60). Interestingly, several viruses from the *Flexiviridae* family that infect seasonal or perennial plants possess nonfunctional or entirely missing vALKB domains, suggesting that this feature may play only a marginal or situational role in viral survival or adaptation (58, 60). Artificially introduced ALKB-*like* domains are rapidly lost in potyviruses, suggesting they offer little evolutionary advantage. In contrast, the introduction of additional PTGS-inhibiting genes, such as *2b*, is preserved, even when redundant with the fully functional HC-Pro protein, the primary PTGS inhibitor in potyviruses (69). Interestingly, among the known members of the potyviral family that possess vALKB domains and infect perennial plants, blackberry virus Y (BVY) has a defective HC-Pro protein. This defect may necessitate the presence of a demethylase to counter the plant’s PTGS-based defenses, potentially explaining the conservation of vALKB domains in BVY (59, 60). Potyviral P1 can influence p-body formation, and co-localizes with them similarly to what was reported for *AtALKB9b* (38, 70). This may be necessary for BVY to successfully rescue methylated vRNAs that are sequestered into p-bodies, allowing the virus to evade plant defense mechanisms. Although further studies are needed to clarify this topic, the presence of conserved demethylase domains in certain viruses, along with the direct interaction between AMV-Cp and cellular demethylases, strengthens the correlation between viral mechanisms and m6A methylation (55, 57, 58, 60). A summary of all the identified viruses containing ALKB-*like* domains can be found in Table 2.

**TABLE 2:** Known viruses harboring vALKB domains^*a*^

Virus	Family	vALKB domain	RefSeq ID	Reference
Grapevine rupestris stem pitting-associated virus	Betaflexiviridae	Functional	NC_001948	Born et al. (60)
Cherry green ring mottle virus	*Betaflexiviridae*	Functional	NC_001946	Born et al. (60)
Cherry necrotic rusty mottle virus	*Betaflexiviridae*	Functional	NC_002468	Born et al. (60)
Cherry mottle leaf virus	*Betaflexiviridae*	Functional	NC_0025004	Born et al. (60)
Apricot pseudo-chlorotic leaf spot virus	*Betaflexiviridae*	Functional	NC_006946	Born et al. (60)
Citrus leaf blotch virus	*Betaflexiviridae*	Functional	NC_003877	Born et al. (60)
Malva mosaic virus	*Alphaflexiviridae*	Functional	YP_667844	Born et al. (60)
Alstroemeria virus X	*Alphaflexiviridae*	Functional	YP_667844	Born et al. (60)
Scallion virus X	*Alphaflexiviridae*	Non-functional	NC_003400	Born et al. (60)
Narcissus mosaic virus strain New Zealand	*Alphaflexiviridae*	Non-functional	AY225449	Born et al. (60)
Indian citrus ringspot virus	*Alphaflexiviridae*	Functional	NC_003093	Born et al. (60)
Papaya mosaic virus	*Alphaflexiviridae*	Functional	NC_001748	Born et al. (60)
Alternanthera mosaic virus	*Alphaflexiviridae*	Functional	NC_007731	Born et al. (60)
Apple stem pitting virus	*Betaflexiviridae*	Functional	NC_003462	Born et al. (60)
Apple stem pitting virus isolate PR1	*Betaflexiviridae*	Non-functional	EU095327	Born et al. (60)
Grapevine virus B	*Betaflexiviridae*	Non-functional	NC_003602	Born et al. (60)
Grapevine virus a isolate PA3	*Betaflexiviridae*	Functional	AF007415	Born et al. (60)
Cactus virus X	*Alphaflexiviridae*	Functional	NC_002815	Born et al. (60)
Zygocactus virus X	*Alphaflexiviridae*	Functional	NC_006059	Born et al. (60)
Opuntia virus X	*Alphaflexiviridae*	Functional	NC_006060	Born et al. (60)
Clover yellow mosaic virus	*Alphaflexiviridae*	Functional	NC_006060	Born et al. (60)
Shallot virus X	*Alphaflexiviridae*	Functional	NC_003795	Born et al. (60)
Chrysanthemum virus B	*Betaflexiviridae*	Functional	NC_009087	Born et al. (60)
Garlic latent virus	*Betaflexiviridae*	Functional	NC_003557	Born et al. (60)
Sweet potato chlorotic fleck virus	*Betaflexiviridae*	Functional	NC_006550	Born et al. (60)
Poplar mosaic virus	*Betaflexiviridae*	Functional	NC_005343	Born et al. (60)
Coleus vein necrosis virus	*Betaflexiviridae*	Functional	NC_009764	Born et al. (60)
Potato rough dwarf virus	*Betaflexiviridae*	Non-functional	NC_009759	Born et al. (60)
Lily symptomless virus	*Betaflexiviridae*	Functional	NC_005138	Born et al. (60)
Blueberry scorch virus	*Betaflexiviridae*	Functional	NC_003499	Born et al. (60)
Potato virus S	*Betaflexiviridae*	Non-functional	NC_007289	Born et al. (60)
Passiflora latent carlavirus	*Betaflexiviridae*	Functional	NC_008292	Born et al. (60)
Phlox virus S	*Betaflexiviridae*	Functional	NC_009383	Born et al. (60)
Phlox Virus B	*Betaflexiviridae*	Functional	NC_009991	Born et al. (60)
Hop latent virus	*Betaflexiviridae*	*Betaflexiviridae*	NC_002552	Born et al. (60)
Daphne virus S	*Betaflexiviridae*	Functional	NC_008020	Born et al. (60)
Aconitum latent virus	*Betaflexiviridae*	Functional	NC_002795	Born et al. (60)
Narcissus common latent virus	*Betaflexiviridae*	Functional	NC_008266	Born et al. (60)
Hippeastrum latent virus	*Betaflexiviridae*	Non-functional	DQ098905	Born et al. (60)
Potato virus M isolate Uran	*Betaflexiviridae*	Non-functional	AY311394	Born et al. (60)
Black raspberry necrosis virus	*Picornavirales*	Functional	NC_008182	Born et al. (60)
Little cherry virus two strain LC5	*Closteroviridae*	Functional	AF416335	Born et al. (60)
Grapevine leafroll-associated virus 3	*Closteroviridae*	Functional	NC_004667	Born et al. (60)
Plum bark necrosis and stem pitting-associated virus	*Closteroviridae*	Functional	NC_009992	Born et al. (60)
Blackberry virus Y	*Potyviridae*	Functional	NC_008558	Born et al. (60)
Endive necrotic mosaic virus	*Potyviridae*	Functional	OM867853	Yue et al. (59)
French Endive necrotic mosaic virus	*Potyviridae*	Functional	KU941946	Yue et al. (59)

^*a*^ List of the known viruses harboring ALKB-*like* domains, grouped by family (The *Flexiviridae* family is divided into *Alphaflexiviridae* and *Betaflexiviridae*, following the most recent taxonomic classification).

Table 2 List of the known viruses harboring ALKB-*like* domains, grouped by family (The *Flexiviridae* family is divided into *Alphaflexiviridae* and *Betaflexiviridae*, following the most recent taxonomic classification).

## LEVERAGING m6A MODIFICATIONS TO ENHANCE VIRAL RESISTANCE IN AGRICULTURE

Viruses are one of the greatest threats to modern agriculture, capable of causing substantial losses across crops, perennial plants, and other cultivars (71–74, 75). Although knowledge of RNA methylation, particularly (m6A), and its downstream effects in plants is still limited, recent studies suggest that alterations in m6A-mediated processes can enhance plant resilience to viruses, potentially leading to viral resistance. Traditional agricultural strategies—such as fertilizers, pesticides, and antibiotics—are largely ineffective against viral infections. However, a deeper understanding of the interplay between viruses and the plant methylome offers the potential for novel solutions. The specificity of m6A-virus interactions means that small mutations in viral or host genomes can dramatically alter viral behavior. For example, single nucleotide polymorphisms (SNPs), as shown by Zhang et al. (16), can significantly reduce a virus’s ability to exploit the host. Modern sequencing technologies enable precise identification of both viral and host genomes, facilitating the search for cultivars harboring resistance genes. By focusing on plants with naturally occurring m6A-related resistance mechanisms, breeders can significantly boost viral resistance. Furthermore, combining m6A-based resistance with other viral resistance pathways can enhance the plant’s defense arsenal. Although absolute viral resistance may be difficult to achieve, the strategic breeding of resistant crops could dramatically reduce product loss in agriculture within a relatively short time.

## CONCLUSIONS

Although the number of studies regarding interactions between plant viruses and m6A are currently limited, recent discoveries are providing additional insights into the mechanisms that govern the interaction between the plant methylome and viruses. The majority of the data gathered so far suggest a significant detrimental impact of m6A methylation on plant viruses, which are summarized in Fig. 2 and 3 using AMV and PepMV as models (38, 53, 54, 62, 63, 65). Nevertheless, within the animal field, it has been demonstrated that RNA viruses can have both advantageous and detrimental interactions with the cell methylome, contingent upon the specific combination of host and virus (47, 48, 50, 51). This was also observed in plants, where TaMTB was actively recruited by WYMV, as represented in Fig. 4 (16) and summarized in Table 1. However, to strengthen the role of m6A in facilitating viral infections in plants needs further studies. In addition, plant viruses can undergo methylation during their transportation by the insect vector (66). Further research is needed to determine whether these alterations specifically affect the relationship between the virus and its vector or if they have any additional impacts on the plant host. Prior studies in animal research successfully identified and defined the specific function of m6A methylation on the viral transcripts being studied (47, 51). When studying plants, this task becomes more difficult due to the larger number of proteins involved in erasing and reading genetic information. Additionally, some plants have duplicated genomes, which means they can have more than two versions of the same gene. Plant viruses can also have functional ALKB-*like* domains, which have never been reported for animal viruses. Although the exact function of these domains remains unclear, the current evidence strongly suggests that they play a role in RNA demethylation (Table 2) (57–60). In the evidence so far collected, there is frequently a direct connection between viral proteins and the host methyltransferase complex, which can be exploited as a target for developing virus-resistant crops (16, 55, 62). In summary, although our understanding of this topic remains limited, the data gathered thus far indicate shared characteristics among various viruses. This knowledge could now be employed to specifically target host genes in order to enhance virus resistance in crops.

### Outstanding questions

- Mechanisms of interaction: Are there common patterns between similar viruses governing the interaction between plant methylome and pathogen?
- Evolutionary adaptation: Were the conserved ALKB domains in the *Flexiviridae* and *Potyviridae* virus families introduced through manmade pressure (e.g., pesticides) or as a natural means to counter m6A-mediated plant defense?
- Methylation patterns: What is the impact of viral infections on the methylation patterns of plant cells, and how much of this is attributed to the plant defense mechanisms versus direct viral influence?
- Detrimental vs. beneficial effects: How does m6A methylation impact plant viruses negatively, and are there instances where it might have a beneficial impact similar to observations in animal viruses?
- Is the localization of the methyltransferases, demethylases, and readers altered by viral infections? And if so, is it the result of a cellular response or is it caused by the pathogen?
- Vector influence: How do methylation alterations during transportation by insect vectors affect the relationship between the virus and its vector, and what additional impacts might these have on the plant host?
- How do plant microbiota shape the host m6A landscape, potentially regulating subsequent viral infections? Additionally, how do non-pathogenic viruses within the natural plant microbiome interact with the host m6A machinery, influencing each other in the process?

By addressing these questions, it will be possible to gain a deeper understanding of the role of m6A methylation in plant-virus interactions and potentially develop strategies to enhance virus resistance in crops.
